# Synergistic effects of bacterial consortium and thermal energy on treatment of sewage by waste stabilisation pond

**DOI:** 10.1016/j.mex.2023.102333

**Published:** 2023-08-18

**Authors:** Ijeoma I. Nwajuaku, Jonah C. Agunwamba

**Affiliations:** a Department of Civil Engineering, Faculty of Engineering, Nnamdi Azikiwe University, Awka, Anambra State, Nigeria; b Department of Civil Engineering, Faculty of Engineering, University of Nigeria, Nsukka, Nigeria

**Keywords:** BOD/COD ratio, Mass concrete, Radiate heat, Temperature, Insulation, Biodegradation, Integrated thermal coating and inoculum method for biodegradation of sewage wastewater

## Abstract

The low rates of biodegradation of organic pollutants in wastewater have been attributed to the daily fluctuation of temperatures, which affects microbial metabolism and activities in reactors.  This work aimed to develop a method to degrade sewage pollutants using a synergistic effect of bacterial consortium and thermal energy, while a grey concrete pond served as the control. The results demonstrated that the temperature profile of ICCP showed that all through the experiment, the temperature was above 25 °C, which is a suitable temperature for mesophilic bacterial growth. A properly-stabilised effluent was achieved by the ICCP with a low biodegradation index between 0.11 and 0.14.  The values of BOD (95%) and COD (74%) removal efficiencies were obtained at a 10-day retention time in ICCP, which is in accordance with standard of the United State Environmental protection Agency. Moreover, a comparison between a control and ICCP revealed that the latter emits heat energy 30% higher than the first. The temperature of 30 °C (dark) and 30.8 °C (light) produced a BOD removal > 90%. Therefore, this method could be considered to bridge the gap in daily fluctuation of temperature for enhanced biodegradation.

• Designing of a thermal coated concrete pond to investigate their thermal performance during the dark and light condition
• Bioremediation test for selection of  mixed bacteria strain of high degradation potential used as   inoculum
• A detention time of 10 days under natural sunlight used for investigation for concentration balance of organic  pollutant

## Graphical abstract

**Figure:**
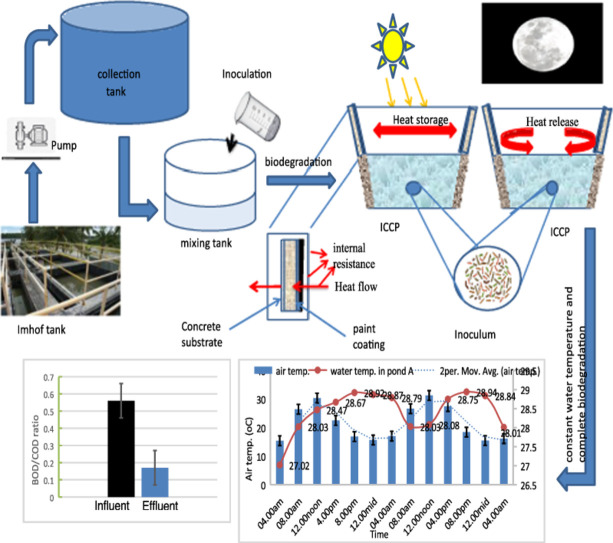

Specifications table

**Table:** 

Subject area:	Environmental Science
More specific subject area:	*Wastewater treatment and combined techniques.*
Name of your method:	*Integrated thermal coating and inoculum method for biodegradation of sewage wastewater*
Name and reference of original method:	*N.A.*
Resource availability:	*All resources are detailed within this article*

## Introduction

Wastewater in the sewage system contains significant thermal energy, with 85% of the energy being heat and the remaining 15% being organic material and nutrients [1]. In countries with temperate climate (Russia, Croatia, and Scandinavia), systems have been used to recover heat from wastewater treatment plant effluent for various applications [2], [3], [4] such as district heating, cooling, agricultural greenhouses, and drying dewatered sludge [5]. However, reducing the inlet temperature of the wastewater treatment plant can reduce treatment efficiency and effluent quality.

Conventional waste stabilisation ponds are natural-based systems that biologically treat wastewater within an extensive hydraulic retention time (HRT) [6], [7], [8]. However, their performance is affected by climatic conditions, such as low air temperature, high temperature variation, and sunlight intensity. Despite the fact that sewage contains highly diverse microbial communities with many degradative capacities, biodegradable synthetic compounds persist in the environment, and inoculation with specific high degraders capable of metabolising chemicals has been proposed as a potential method. The integration of physicochemical conditions and biological processes can speed up the decomposition of organic matter by bacterial consortia [9,10].

Thermal coating, which involves using thermal paint to modify substrate properties, is a useful method for heat energy conservation in structures. Thermal paints reinforced with ceramic-based microspheres achieve thermal insulation [11], and black pigments have high absorption and emissivity at high temperatures [12]. In this work, the integrated system of bacteria consortium and thermal energy for wastewater treatment is investigated.

## Methods

This sewage treatment method is based on the inoculation (with the bacteria consortium) and insulation (conservation of thermal energy) the of the treatment pond. The modification of the thermal properties of concrete (substrate) by coating, for improved thermal efficiency was achieved via the optical properties of thermal paint.

### Characterisation of thermal coating

Isonem thermal paint is water-based and elastomeric resin-based, with vacuum microspheres manufactured by Isomen Paint and Insulation Technologies Turkey. The thermal properties include high solar absorbency, high surface heat transmission value and low thermal conductivity as shown in Table 1(a). Other technical specifications are given in Table 1(b).

**Table 1a:** Thermal properties of thermal paint used in the study (Isomen Paint and Insulation Technologies, 2015).

Sunlight absorbency value (α)	Surface heat emissitivity value (ε)	Thermal surface resistance (RS)	Heat conductivity coefficient (W/mK)	Heat Conductivity (λ)
0.82	0.80	0.0495 ± 1.5%	0.023	<0.060

**Table 1b:** Technical specification parameters ( Isomen Paint and Insulation Technologies, 2015).

pH	Density (g/mL)	Viscosity ( mpa.s)	Water transmission rate (kg/m^2^. .h^0.5^)	Adhesion strength (N/mm^2^)	Water vapour Permeability(m)
7.0 – 9.0	0.85±0.10	12,500 - 13,500	<0.1	≥0.8	5≤ S_D_≤ 50

### Settled sewage collection

Settled sewage was abstracted using a 5.5 Hp water pump from a settling unit of the sewage treatment plant located at the University of Nigeria, Nsukka (UNN). The sewage was channelled to the pilot-scale concrete ponds designed and constructed for this study. The characteristics of the sewage are shown in Table 2.

**Table 2:** Physical and chemical properties of the settled sewage used in this study.

	pH	Temp (°C)	DO (mg/l)	BOD	COD	TSS	NO_3_- N
Settled sewage	7 ± 07	25 ± 3	4 ± 2	50 ± 3	123 ± 2	26 ± 2	1.08 ± 0.2

### Isolation of microbial consortium

Using a serial dilution approach, ten bacterial strains labelled strain No.1 to strain No.10 (S1-S10) were obtained from UNN's facultative pond. A ten-fold serial dilution of the sample was made, from which 0.1 ml was plated into a sterile Petri dish. Using pour plating procedures, Nutrient Agar (NA) (Titan Biotech) was produced according to the manufacturer's specifications and poured onto sterilised Petri dish containing 0.1 ml of the sample. The plate was then incubated at 37 °C for 24 h to produce a mixed culture. Isolated colonies were recorded and purified to obtain pure culture by repeated sub-culturing on fresh media for primary isolation, as described by Zhang et al. [13]. Each isolated bacteria was subjected to a bioremediation screening study, with BOD and COD values measured every 24 h for 5 days. The three most efficient strains (S1, S7, and S10) were selected and combined based on their strong degrading capabilities and removal efficiency of the contaminants. After no zone of inhibition was identified at the point of intersection of the isolates, the bacterial consortium was adopted as inoculum.

### Inoculum preparation

The Bacteria consortium was composed of bacteria of the genus *Bacillus spp*. and each bacterium was inoculated separately in Trypticase Soy Broth and incubated at 37 ^o^C for 24 h. After that time, each culture was centrifuged at 12,000 rpm for 10 min, and the cell was washed twice in sterile saline. Then the washed bacteria strain was resuspended in sterile normal saline, and microbial suspensions were adjusted in nephelometer tubes to 0.5 McFarland standards (optical density of 0.1 at 620 nm) with sterile normal saline water. Then, these suspensions were mixed in equal proportions to obtain a suspension with a final concentration of viable cells of 1 × 108 cell/mL

### Preparation of concrete coated pond

Three coated concrete ponds were built as plain cement concrete (grade M15) according to India Standard code of practices (IS 456–2000). The specification for the concrete mix design is shown in Table 3. The ponds were cured for 28 days at room temperature. Then, using a wall brush, a layer of Isonem universal primer (1:7 diluted with water) was applied to the concrete interior surfaces and allowed to dry for 4 h before applying the thermal black paint (ISO 321). Three thin coats of paint (1 mm thickness) were applied at 4 hour intervals to obtain a dry and homogenous surface under ambient temperature for 72 h. This ensures that there is sufficient curing before usage to avoid thermal fracture.  All the ponds were built following different length-to-width ratios, but with the same measurement of the freeboard and wall thickness. These ponds were designated ICCP-A, B and C as shown in Table 4.

**Table 3:** Specification for concrete mix design.

M15 ratio	1:2:4
Portland cement	33 Grade(IS:12,269)
Natural river sand	4.75mm
Crushed granite aggregate	40mm(IS:383)
Water absorption	0.20%
pH of portable water	7.7
Water-cement ratio	0.40
Concrete placement	By hand

**Table 4:** Pond sizes, aspect ratio and flow area of the model ponds.

Exp. Pond	Size(m)	Aspect ratio	Surface (m^2^) area	Water volume (m^3^)	Water depth (m^3^)
A	1.5 × 0.38 × 0.61	4	0.570	0.2907	0.51
B	1.5 × 0.30 × 0.61	5	0.450	0.2295	0.51
C	1.5 × 0.25 × 0.61	6	0.375	0.1913	0.51

All ponds were designed with the same freeboard of 0.9 m, and a wall thickness of 0.1016 m.

### Preparation of evaluation system

**Fig. 1:**
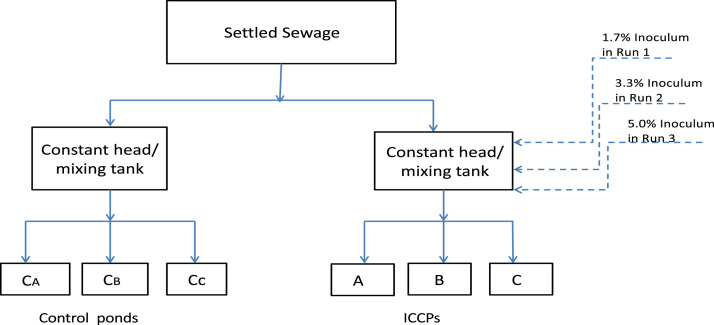
The flow chart of the treatment process.

In this study, the experimental set-up consisted of six rectangular concrete ponds; three ponds were coated (ICCP), and the other three were uncoated (control). This set of control ponds (CA, C_B_ and Cc) were duplicates of ICCPs. All ponds were exposed to both light and dark conditions. In the treatment process, the mixing tank (0.18 m^3^) was operated at a constant head and fed with settled sewage from an overhead tank (4 m^3^). Then the inoculum dosages of 1.7, 3.3, and 5.0% (v/v) were added to the tank at different experimental runs. After inoculation, a 48-hour acclimation period was allowed for bacteria growth. Then, the sewage was gravitationally moved to the ICCPs via a 25-mm branched pipe. Each experimental run was performed in triplicate. Ponds A, B, and C operated at a hydraulic loading rate of 80.92 m^3^/d/m^2^, 62 m^3^/d/m^2^, and 58 m^3^/d/m^2^ respectively, with a final total flow rate of 120.96 m^3^/d.  The results were compared with those of the control, where the mixing tank was not inoculated. The flow chart of the treatment process is shown in Fig. 1.

### Molecular identification

Genomic DNA was extracted from strains 1, 7, and 10 using the quick DNA Fungal/Bacteria Miniprep kit (Zymo Research Catalogue No. D6005). The 16S target region was amplified using One Taq Quick-load 2X Master Mix (NEB, Catalogue No. M0486) with the following primers: 27f(AGAGTTTGATCMTGGCTCAG) and 1492r (CGGTTACCTTGTTACGACTT $\overset{\prime }{3}$). After purification, the PCR product was identified by Iqaba Biotech West Africa Ltd. The similarity of species obtained in this study with other species was checked using the BLAST program at www.ncbi.nlm.nih.gov. MEGA11 software was used to calculate genetic distances. The morphological, physiological, and biochemical features of the bacterial strain were identified at 37 ± 1 °C under a microscope, and the preliminary identification was done based on the results of the physiological and biochemical features of the bacterial strains. The sequencing analysis showed that S1 is GT-5 (Gen Bank accession number MT492023.1) with a high degree of nucleotide identity (99.18%), S7 is LSB 7 (GenBank accession number MK600526.1) with 95.39% identity, and the sequence of S10 is fort 87 (GenBank accession number MG561362.1) with 99.52% identity. Therefore, S1, S7, and S10 were all identified as Bacillius cereus.

### Determination of a physicochemical parameter

At every 24 h, 50 mL samples were taken from each pond for the determination of the following parameters: biochemical oxygen demand (BOD) and chemical oxygen demand (COD); dissolved oxygen-Azide modification of the Winkler method; and dichromate reflux method. Total organic carbon (TOC): high-temperature catalytic oxidation method, by APHA Standard Methods [14]. Measurement of dissolved oxygen (mg/L) was taken with a JPB-70 pen-type intelligent dissolve oxygen analyser (range 0.0 to 20 mg/L, 0.0 to 4.0 °C). Surface and water temperatures were taken with an infrared thermometer (Testo 830-T2 IR) with a connectable temperature probe (TC type K) (range −50 to +500 °C, resolution 0.1 °C, and accuracy  ± 0.5). The hydraulic retention time was 10 days, and three replicates of each experiment were carried out.

### Determination of surface and water temperatures of the treatment system

All ponds were operated in a daily fed-batch mode on a sunny day, at a hydraulic retention time (HRT) of 48 h. During this period, the changes in water temperature were monitored hourly, until a steady-state heat transfer was reached (at a water temperature above 28 °C). Once this was achieved, wastewater was sampled at 4-hourly intervals for two consecutive days, and the 4-hourly mean air and wastewater temperatures were measured. Then, the surface temperatures of the ICCP were recorded over 8 h to determine the maximum temperature for the thermal coating.

### Determination of energy emitted by the thermal systems

The determination of energy balance is for important for making accurate assumptions on the thermal behaviour of insulated pond system in order to achieve improved energy efficiency. All calculations were made using absolute temperature (Kelvin scale).

### Commonly used symbols

ε   emissivity

σ  Stefan-Boltzmann constant [5.670367 × 10^–8^ ] W/m^2^/K^4^

T     temperature [K]

E_b_   Energy emitted by a black body [J]

E     Energy emitted by a real body [J]

The energy (E) emitted by a body was calculated using Eqs. (1) and (2) [15].

(1) $$E_{b}\left(T\right)=\varepsilon \sigma T^{4}$$

(2) $$\varepsilon \left(T\right)=\;\frac{E\left(T\right)}{E_{b}\left(T\right)}$$

The equations for the radiative energy released by a certain body have a direct relationship with three essential concepts: emissivity, grey body, and black body. The black and grey bodies are idealised representations of Planck's radiation law. The former is a perfect emitter with a hemispherical total emissivity value of one (ε = 1), whereas the other is a body that emits with less intensity. It is important to note that the emissivity (ε), which measures how much energy a substance emits relative to a black body (a perfect emitter), has a value that ranges from 0 to 1:

In order to evaluate the thermal performance of the uncoated concrete (control), surface temperature measurements were taken for 1 week under sunny days. Then, using Eq. (3), which describes the thermal balance of a surface under the sunlight emissivity of the control was calculated as follows [16]:

(3) $$\left(1-a\right)l=\varepsilon \sigma \left(Ts^{4}-{T^{4}}_{\text{sky}}\right)+\text{hc}\left(\text{Ts}-\text{Ta}\right)$$

where:

a solar reflectance or albedo of the surface

I total solar radiation incident on the surface (*I* = 1000 W/m^2^)

ԑ emissivity of the surface (adopt 0.39)

σ Stefan-Boltzmann constant, 5.6685 × 10^–8^ Wm^-2^ K^-4^

Ts surface temperature, K

Tsky the effective radiant sky temperature (Tsky =300 K)

hc convection coefficient, (hc =12 Wm^-2^ K^-1^ )

Ta air temperature, (Ta =310 K)

The energy emitted by the control was computed using Eq. (4).

(4) $$E\left(T\right)=E_{b}\left(T\right)\times \varepsilon \left(T\right)$$

Sands, cement hydration products, and aggregates make up the majority of a concrete mix. Thus, the albedo of a sample of concrete is the sum of the albedos of the three materials mentioned above. Concrete's albedo is between 0.35 and 0.40 when it is newly hardened, and it drops to between 0.20 and 0.30 as it ages and weathers [17].

### Cost evaluation

The expenditure for the production of 1m^3^ of concrete was presented in Tables 5 and 6.   The construction cost for wastewater treatment plants vary considerably based on the price of land in different locations, with yearly maintenance expenditure of a particular percentage of the construction cost.

**Table 5:** Market price of cost of production of 1m^3^ of concrete.

Concrete materials	unit	Unit cost ($)	Volume	Total price($)
Cement	kg	0.107	350	37.45
Sand	m^3^	11.32	0.44	4.98
Aggregate	m^3^	34.68	0.88	30.52
Water	m^3^	1.11	0.18	0.20

Total				73.15

**Table 6:** Cost of construction of 1m^3^concrete coated pond.

Total material cost	$73.15
Cost of thermal paint and primer	$6.45
Labour output (placing of concrete and coating)	$11.1
Total cost of production	$177.4

### Determination of removal efficiency

The removal efficiency of organic pollutant such as BOD or COD was evaluated according to retention time in the treatment pond and was calculated as:

(5) $$\;Percentage\;removal\;efficiency=\;\frac{BOD_{i}-BOD_{e}}{BOD_{i}}\times 100$$

Where:

BODi     Influent BOD

BODe     Effluent BOD

## Method validation

### BOD and COD removal efficiency in ICCP and control C

**Fig. 2:**
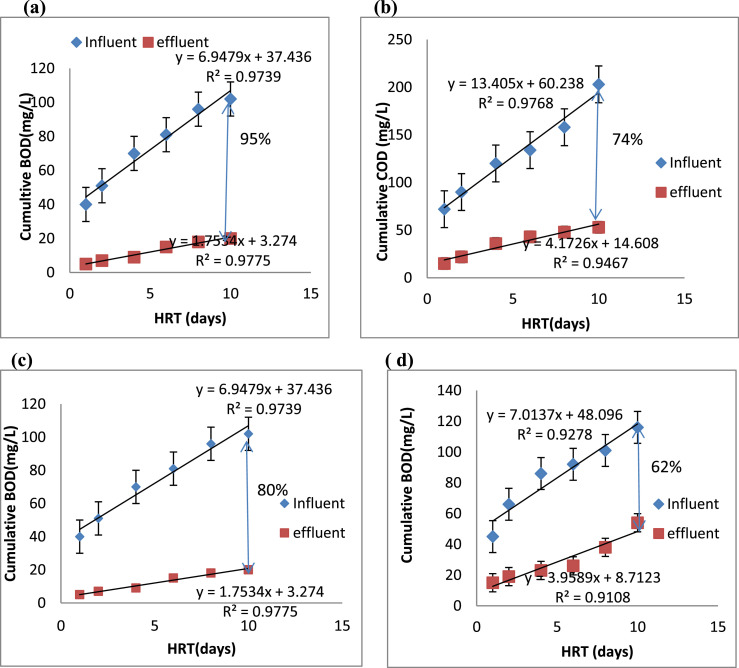
Influent and effluent balance for BOD and COD in (a) & (b) ICCP  and (c) & (d) control pond.

The performance of ICCP was tested by estimating the organic matter removal efficiency over 10 days. The graphs obtained from the cumulative concentration at the inlet and outlet of the ponds at the optimum inoculum (3.3%) is shown in Fig. 2. The BOD removal efficiency of 95% was obtained from ICCP with 15%, higher than that of the control, indicating that thermal coating played a role in achieving maximum biodegradation through increased temperature. This was attributed to vacuum microspheres (a special additive insulate) contained in the thermal paint, responsible for reducing heat loss from the pond [18].  For the COD parameter, the percentage of removals from final effluents was moderate in the control (62%) and high in the ICCP (74%). The COD removal efficiency may depend on the type of microorganism and the level of their activities [19].

**Fig. 3:**
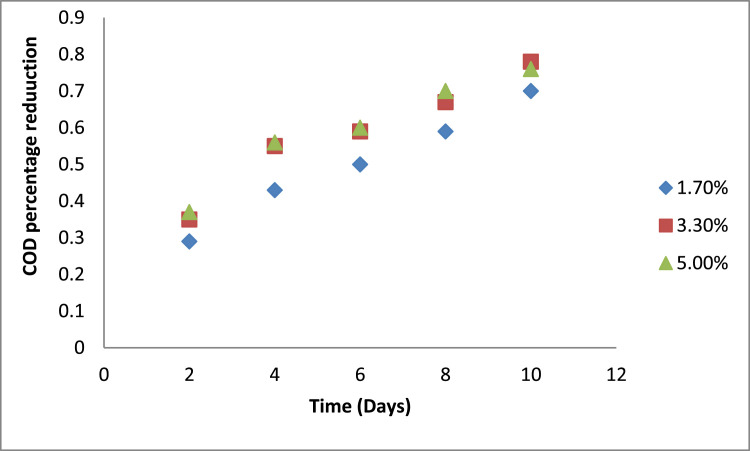
Graph of COD reduction percentage over time with the use of  three inoculum dosages.

Fig. 3 shows the percentage reduction in COD, where the treatments with the highest percentage of inoculum (5%) maintained a high efficiency from day 2 - day 8, and yielded an insignificant increase in the COD percentage reduction compared to 3.3%;. However, 3.3% bacterial inoculum achieved a maximum reduction in the COD reduction values on day 10, which indicated a better performance in the organic biodegradation process. This indicated that the 3.3% dosage is the optimal dose in consideration to the economical aspect of the treatment process.

### Biodegradability index between the integrated and control ponds

The biodegradability index reveals that the BOD/COD ratio of the ICCP and control ranged from 0.11 to 0.14 and 0.33–0.45 respectively. A study reported that a complete biodegradation was achieved, with a BOD/COD ratio range of 0.11–0.13 [20,21]. The control, having a ratio within 0.3–0.6, displayed an incomplete degradation, which is an attribute of a low rate of biodegradation [22]. Organic matter  degradation is influenced by water temperature, as seen by the control pond, which fluctuated between the low (15 °C–19 °C) and intermediate (20 °C–24 °C) ranges, while a high- temperature range of 25 °C-30 °C, was attained by ICCP. The temperature was attributed to the rapid degradation rate in the ponds, indicating that the coating increased the heat absorbability of the concrete on sunny days, and maintained a stable water temperature for microbial activities at night. The incomplete and complete biodegradation were observed at 24 °C and 30.1 °C in the control and ICCP respectively. Hence, the increased in temperature resulted in low BOD/COD ratio.  This was due to the stability in the temperature (above 24 °C); an optimum condition for biological activity of mesophilic bacteria. The biodegradability index for the influent and effluent from ICCP and  control was depicted in Table 7.

**Table 7:** Comparison of Biodegradation index of sewage between ICCP (at 3%v/v) and control.

Run	Influent	Temp.(°C)	ICCP Effluent	Temp.(°C)	Control Effluent
1	0.88±0.15	29.6	0.13±0.05	19.3	0.45±0.04
2	0.77±0.11	26.8	0.14±0.03	21.0	0.37±0.03
3	0.81±0.12	28.0	0.13±0.02	20.9	0.39±0.02
4	0.89±0.14	28.2	0.12±0.02	18.6	0.42±0.01
5	0.95±0.13	29.5	0.12±0.01	22.8	0.37±0.01
6	0.80±0.12	30.1	0.11±0.03	24.2	0.33±0.01

### Effect of radiant heat storage on pond water temperature

**Fig. 4:**
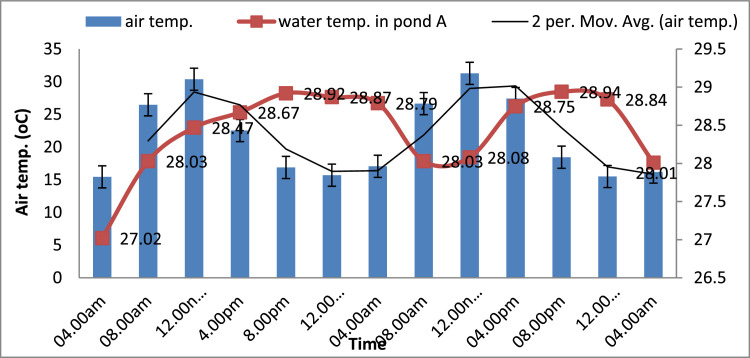
Average Water and air temperature variation in the ICCP at 48 h duration.

Fig. 4 shows a wide fluctuation of air temperature from a high temperature of 30.38 °C near midday to a low temperature of 15.7 °C in the middle of the night. On the other hand, the water temperature increased from 28.0 ^o^C to 28.67 °C during a period of 8.00 am to 4.00 pm. A high-water temperature of 28.92 °C was attained at 8.00 pm; at a time heat was being conserved in the water. Extra heat energy (28.87 °C) was transferred to the water through convection from 4 pm - 4.00 am of the next morning. As expected, the temperature at 4.00 pm was higher compared to the temperature that occurred at noon. This is attributed to the sufficient heat stored within the peak hours of solar radiation. A research [23] has reported the possibility of a proportional increase in solar irradiation with time, and could attain its intensity during the midday between 12:00–14:00 (peak hours).

The thermal mass effect also produced a time lag, where there was a delay in the time of peak temperature, which occurred 8 h (from 12 noon to 8 pm) later than the peak air temperatures. The time lag may be due to a low thermal mass diffusivity of concrete; a delay caused by certain properties of the concrete such as density, thickness, specific heat and surface absorptivity. The water temperature displayed a similar trend of an increase and a decrease in different locations compared to the air temperature. Hence, heat absorbability was assessed based on the surface temperature of the concrete. The concrete surface absorptance has been reported to be 60% and could increase as high as 96% when it is black-painted [24].

**Fig. 5:**
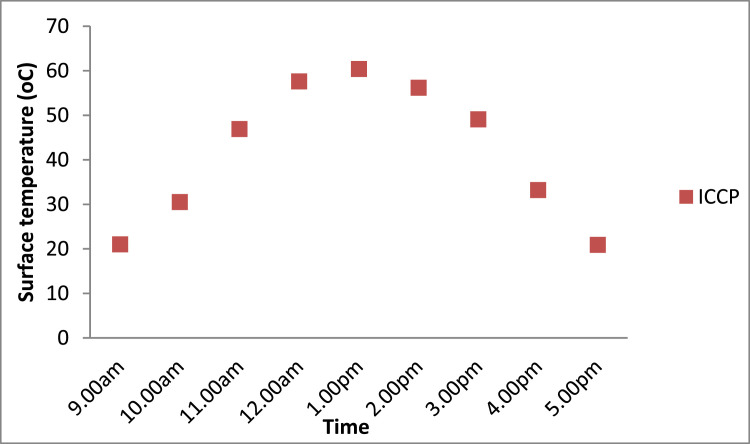
Curve showing the temperature limits of the thermal coating.

The actual performance of the thermal coating is demonstrated in Fig. 5. The surface temperature of the ICCP was recorded over 24 h on a sunny day. During the morning hours from 00:00 to 09:00, the temperature of ICCP was below 25 °C.  From 09:00 to 14:00; when the samples were in direct sunlight, the temperature attained a maximum temperature of 60.4 °C. After 14:00, there was reduction in solar intensity, though the rest of the sky was mostly clear. This condition persisted for a few hours until there was a drop in the surface temperature below 25 °C; which was similar to the temperature that occurred during 00:00 to 09:00 period. The curve depicts the temperature limits of the thermal coating.

Table 8 reveals the importance of the type of coating suitable for wastewater application. The black colour of the coating had emissivity equal to 0.80, which is slightly higher than the grey colour of concrete (0.7). The difference in the emissivity of these two colours might seem insignificant, but the results showed that the ICCP was more effective in releasing higher amount of heat energy during the day compared to the control. In other words, the thermal coating was proved to a better heat source, which could effectively emit the radiant heat and at the same time, slow down the heat flow to the outer surface of the concrete wall as well.

**Table 8:** Surface temperatures and energy emitted by the colours of the ICCP and control.

Ponds	ԑ	σ (W/m^2/^K^4^)	Ts (K)	E_b_ (J)
ICCP	0.80	5.670367 × 10^–8^	333.40	560.48
Control	0.70	5.670367 × 10^–8^	312.53	392.34

## Conclusion

The improved biodegradation of organic matter was achieved through elevated temperature due to the synergistic effect of thermal energy and bacteria consortium. The temperature of 30 °C (dark) and 30.8 °C (light) produced a BOD removal > 90%. The fact is that bacteria enzymes have an optimum temperature at which they are active, and the paint coating was responsible for heat conservation, which in turn; stabilised the water temperature. The inoculum dosage of 5% resulted in an insignificant removal efficiency, which could be considered a waste of time and resources. From the economic implications, 3.3% was an optimum dose of inoculum bacteria capable of enhancing the efficiency of the treatment ponds.   Hence, this novel design is suitable for harmonising diurnal temperature in WSP.

However, one of the main economic challenges of this study is the operating cost associated with the isolation of inoculum, and possibility of inoculum overdose with consequent waste of resources and time. Due to the duration of this experimental study and the conceptualisation of synergistic effects of the two processes, this study has potential limitations. The test conducted focused mainly on the combined effect of the thermal energy conservation and bacteria inoculum on organic removal in WSP at 10 days HRT. As such, further research on the lifespan of the thermal coating under an extensive HRT. Also, the biodegradability rate of organic matter contained in domestic and industrial wastewater is recommended.

## CRediT authorship contribution statement

**Ijeoma I. Nwajuaku:** Methodology, Formal analysis, Investigation, Writing – original draft, Writing – review & editing, Conceptualization. **Jonah C. Agunwamba:** Conceptualization, Methodology, Writing – review & editing, Supervision.

## Declaration of Competing Interests

The authors declare that they have no known competing financial interests or personal relationships that could have appeared to influence the work reported in this paper.

## Data Availability

Data will be made available on request.
